# Supplementary material for: DeepCob: precise and high-throughput analysis of maize cob geometry using deep learning with an application in genebank phenomics
Source: Plant Methods. 2021 Aug 21;17:91. doi: 10.1186/s13007-021-00787-6 (PMC8379755; doi:10.1186/s13007-021-00787-6)
Supplement: Supplementary file 1 — Additional file 1. Additional Tables and Figures. [file 13007_2021_787_MOESM1_ESM.pdf]

DeepCob: Precise and high-throughput analysis of maize cob geometry using deep learning with an application in genebank phenomics

Lydia Kienbaum[1], Miguel Correa Abondano[1], Raul Blas[2], Karl Schmid[1,3]

[1]Institute of Plant Breeding, Seed Science and Population Genetics, University of Hohenheim, Stuttgart, Germany

[2]Universidad National Agraria La Molina (UNALM), Lima, Peru

[3]Computational Science Lab, University of Hohenheim, Stuttgart, Germany

Corresponding author:

Karl Schmid

Email: karl.schmid@uni-hohenheim.de

Supplementary tables

Supplementary Table S1: Training results of the 90 *Mask R-CNN* models. AP@[.5 : .95] scores of the 90 models with different training parameters, for training sets of 200 and 1000 images.

Model	200 images	1000 images	Model	200 images	1000 images
M01	54.60	57.52	M101	86.56	79.67
M02	57.74	38.06	M102	84.68	81.14
M03	67.95	46.42	M103	79.72	76.17
M04	65.64	44.12	M104	86.74	80.02
M05	63.77	52.10	M105	81.92	79.14
M06	29.96	50.78	M106	79.39	78.81
M07	55.21	52.76	M107	86.56	69.68
M08	59.18	66.05	M108	83.89	80.05
M09	69.08	21.60	M109	79.95	76.98
M11	46.38	45.70	M111	81.18	78.88
M12	73.69	55.21	M112	73.76	77.21
M13	40.08	46.51	M113	74.94	75.19
M14	25.75	44.06	M114	84.80	83.78
M15	41.84	46.67	M115	84.80	78.02
M16	48.73	59.71	M116	81.91	77.96
M17	68.58	34.37	M117	84.24	77.25
M18	51.54	56.15	M118	83.43	76.56
M19	59.78	61.71	M119	82.80	81.56
M21	61.83	54.36	M121	80.75	80.28
M22	58.53	65.13	M122	78.59	75.54
M23	48.76	10.49	M123	68.42	73.25
M24	69.17	38.14	M124	86.56	79.10
M25	62.00	21.68	M125	84.83	78.60
M26	63.47	29.07	M126	81.89	79.21
M27	41.71	38.36	M127	82.00	79.74
M28	75.96	43.72	M128	85.87	79.04
M29	59.29	64.24	M129	62.80	79.84
M31	28.11	54.18	M01	83.09	81.76
M32	63.71	37.46	M01	82.36	83.13
M33	51.76	52.95	M01	73.26	73.81
M34	68.33	43.32	M01	86.13	78.73
M35	72.48	50.09	M01	84.78	79.35
M36	50.48	66.79	M01	81.95	81.86
M37	58.66	35.87	M01	85.23	79.60
M38	47.44	54.04	M01	84.87	78.91
M39	44.80	60.39	M01	82.71	83.42
M41	5.57	57.13	M131	77.30	76.84
M42	52.13	54.41	M132	76.41	57.45
M43	35.00	56.35	M133	53.91	70.78
M44	52.04	30.45	M134	84.86	84.31
M45	42.20	43.72	M135	83.74	75.36
M46	NA	34.52	M136	74.29	75.66
M47	43.45	56.20	M137	85.03	78.15
M48	30.75	32.01	M138	76.27	83.45
M49	31.50	62.78	M139	69.52	70.62

Supplementary Table S2: P-values of lsmeans for Mask R-CNN model parameter *minimask* with parameter values *yes* or *no*.

Contrast	Estimate	SE	df	t ratio	p-value
no - yes	1.4	0.07	170	19.61	$<$0.0001

Supplementary Table S3: P-values of lsmeans for Mask R-CNN model parameters *trainingdata* $\times$ *epochsoverall*. Parameter values for training data are 200 or 1,000 images, and for epochsoverall 15, 20, 200.

Contrast	Estimate	SE	df	t ratio	p-value
200 images; 15 epochs vs. 200 images; 50 epochs	0.36	0.13	170	-4.39	0.0003
200 images; 15 epochs vs. 200 images; 200 epochs	0.33	0.16	170	-4.42	0.0003
1000 images; 50 epochs vs. 200 images; 200 epochs	0.41	0.13	170	-2.96	0.0407
1000 images; 15 epochs vs. 200 images; 200 epochs	0.38	0.16	170	-2.95	0.0413
1000 images; 50 epochs vs. 200 images; 50 epochs	0.44	0.09	170	-2.77	0.0682
1000 images; 15 epochs vs. 200 images; 50 epochs	0.42	0.13	170	-2.61	0.0995
200 images; 15 epochs vs. 1000 images; 50 epochs	0.42	0.13	170	-2.48	0.1359
1000 images; 200 epochs vs. 200 images; 200 epochs	0.4	0.16	170	-2.47	0.1391
200 images; 50 epochs vs. 1000 images; 200 epochs	0.57	0.13	170	2.02	0.3334
200 images; 15 epochs vs. 1000 images; 200 epochs	0.42	0.16	170	-1.98	0.3553
1000 images; 15 epochs vs. 200 images; 15 epochs	0.56	0.16	170	1.51	0.6584
200 images; 50 epochs vs. 200 images; 200 epochs	0.47	0.13	170	-1	0.9169
1000 images; 15 epochs vs. 1000 images; 50 epochs	0.48	0.13	170	-0.66	0.9861
1000 images; 15 epochs vs. 1000 images; 200 epochs	0.48	0.16	170	-0.48	0.9967
1000 images; 50 epochs vs. 1000 images; 200 epochs	0.5	0.13	170	0.07	1

Supplementary Table S4: Variability of image properties specifically between ImgOld and ImgNew, which were caused only by different imaging conditions. The images taken in ImgNew were generally more standardized than in ImgOld.

Source of variation	ImgOld	ImgNew	Examples
Image resolution	variable resolution	standardized resolution	
Background color	diverse	dark blue only	
Incomplete cobs	present	absent	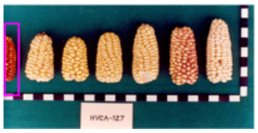
Ruler scale horizontal	2 cm	1 cm	
Ruler scale vertical	2 cm	0.5 cm	
Ruler type	unstacked	stacked	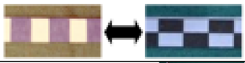
Figure orientation	landscape	landscape or portrait	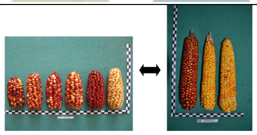

Supplementary Table S5: Number of populations and respective environment represented per department in ImgOld and ImgNew.

		Number of accessions	
Maize landrace	Environment	ImgOld	ImgNew
Rainforest	Amazonas	23	10
	Huanuco	98	28
	Loreto	29	31
	M.Dios	31	33
	San Martin	89	105
	Ucayali	29	20
Highland	Ancash	316	259
	Apurimac	175	158
	Ayacucho	79	128
	Cajamarca	75	34
	Cusco	111	80
	Huancavelica	73	78
	Junin	85	160
	Pasco	25	20
	Puno	14	12
Coast	Arequipa	80	68
	Ica	50	27
	Lambayeque	120	99
	Libertad	113	93
	Lima	58	54
	Mgua	8	5
	Piura	91	88
	Tacna	39	19
	Tumbes	16	10
	Total	1827	1619

Supplementary Table S6: Parameter combinations of the 90 trained *Mask R-CNN* models. 90 trained *Mask R-CNN* models differing in the parameters *learningrate*, *epochs.m*, *epochsoverall*, *masklossweight* and *monitor*. Each model was repeated with and without the use of a mini mask (*minimask*).

model	*learningrate*	*epochsoverall*	*epochs.m*	*masklossweight*	*monitor*	*minimask*
M01	1e-3	200	1	10	val loss	yes
M02	1e-4	200	1	10	val loss	yes
M03	1e-5	200	1	10	val loss	yes
M04	1e-3	200	3	10	val loss	yes
M05	1e-4	200	3	10	val loss	yes
M06	1e-5	200	3	10	val loss	yes
M07	1e-3	200	2	10	val loss	yes
M08	1e-4	200	2	10	val loss	yes
M09	1e-5	200	2	10	val loss	yes
M11	1e-3	50	1	1	mask val loss	yes
M12	1e-4	50	1	1	mask val loss	yes
M13	1e-5	50	1	1	mask val loss	yes
M14	1e-3	50	3	1	mask val loss	yes
M15	1e-4	50	3	1	mask val loss	yes
M16	1e-5	50	3	1	mask val loss	yes
M17	1e-3	50	2	1	mask val loss	yes
M18	1e-4	50	2	1	mask val loss	yes
M19	1e-5	50	2	1	mask val loss	yes
M21	1e-3	50	1	1	val loss	yes
M22	1e-4	50	1	1	val loss	yes
M23	1e-5	50	1	1	val loss	yes
M24	1e-3	50	3	1	val loss	yes
M25	1e-4	50	3	1	val loss	yes
M26	1e-5	50	3	1	val loss	yes
M27	1e-3	50	2	1	val loss	yes
M28	1e-4	50	2	1	val loss	yes
M29	1e-5	50	2	1	val loss	yes
M31	1e-3	50	1	10	val loss	yes
M32	1e-4	50	1	10	val loss	yes
M33	1e-5	50	1	10	val loss	yes
M34	1e-3	50	3	10	val loss	yes
M35	1e-4	50	3	10	val loss	yes
M36	1e-5	50	3	10	val loss	yes
M37	1e-3	50	2	10	val loss	yes
M38	1e-4	50	2	10	val loss	yes
M39	1e-5	50	2	10	val loss	yes
M41	1e-3	15	1	10	val loss	yes
M42	1e-4	15	1	10	val loss	yes
M43	1e-5	15	1	10	val loss	yes
M44	1e-3	15	3	10	val loss	yes
M45	1e-4	15	3	10	val loss	yes
M46	1e-5	15	3	10	val loss	yes
M47	1e-3	15	2	10	val loss	yes
M48	1e-4	15	2	10	val loss	yes
M49	1e-5	15	2	10	val loss	yes
M101	1e-3	200	1	10	val loss	no
M102	1e-4	200	1	10	val loss	no
M103	1e-5	200	1	10	val loss	no
M104	1e-3	200	3	10	val loss	no
M105	1e-4	200	3	10	val loss	no
M106	1e-5	200	3	10	val loss	no
M107	1e-3	200	2	10	val loss	no
M108	1e-4	200	2	10	val loss	no
M109	1e-5	200	2	10	val loss	no
M111	1e-3	50	1	1	mask val loss	no
M112	1e-4	50	1	1	mask val loss	no
M113	1e-5	50	1	1	mask val loss	no
M114	1e-3	50	3	1	mask val loss	no
M115	1e-4	50	3	1	mask val loss	no
M116	1e-5	50	3	1	mask val loss	no
M117	1e-3	50	2	1	mask val loss	no
M118	1e-4	50	2	1	mask val loss	no
M119	1e-5	50	2	1	mask val loss	no
M121	1e-3	50	1	1	val loss	no
M122	1e-4	50	1	1	val loss	no
M123	1e-5	50	1	1	val loss	no
M124	1e-3	50	3	1	val loss	no
M125	1e-4	50	3	1	val loss	no
M126	1e-5	50	3	1	val loss	no
M127	1e-3	50	2	1	val loss	no
M128	1e-4	50	2	1	val loss	no
M129	1e-5	50	2	1	val loss	no
M131	1e-3	50	1	10	val loss	no
M132	1e-4	50	1	10	val loss	no
M133	1e-5	50	1	10	val loss	no
M134	1e-3	50	3	10	val loss	no
M135	1e-4	50	3	10	val loss	no
M136	1e-5	50	3	10	val loss	no
M137	1e-3	50	2	10	val loss	no
M138	1e-4	50	2	10	val loss	no
M139	1e-5	50	2	10	val loss	no
M141	1e-3	15	1	10	val loss	no
M142	1e-4	15	1	10	val loss	no
M143	1e-5	15	1	10	val loss	no
M144	1e-3	15	3	10	val loss	no
M145	1e-4	15	3	10	val loss	no
M146	1e-5	15	3	10	val loss	no
M147	1e-3	15	2	10	val loss	no
M148	1e-4	15	2	10	val loss	no
M149	1e-5	15	2	10	val loss	no

Supplementary Table S7: P-values of lsmeans in the statistical analysis of model updating with the factor dataset:model

Contrast between models	Estimate	SE	df	t ratio	p value
10 images COCO vs. 20 images COCO	-6.517	0.79	944	-8.294	$<$0.0001
10 images COCO vs. 30 images COCO	-6.472	0.79	944	-8.237	$<$0.0001
10 images COCO vs. 40 images COCO	-10.193	0.79	944	-12.973	$<$0.0001
10 images COCO vs. 50 images COCO	-12.345	0.79	944	-15.712	$<$0.0001
10 images COCO vs. 10 images Maize	-17.209	0.78	944	-22.015	$<$0.0001
10 images COCO vs. 20 images Maize	-16.841	0.78	944	-21.519	$<$0.0001
10 images COCO vs. 30 images Maize	-18.286	0.78	944	-23.366	$<$0.0001
10 images COCO vs. 40 images Maize	-18.919	0.78	944	-24.174	$<$0.0001
10 images COCO vs. 50 images Maize	-18.862	0.78	944	-24.060	$<$0.0001
20 images COCO vs. 30 images COCO	0.045	0.79	944	0.058	$<$0.0001
20 images COCO vs. 40 images COCO	-3.676	0.79	944	-4.679	$<$0.0001
20 images COCO vs. 50 images COCO	-5.828	0.79	944	-7.417	$<$0.0001
20 images COCO vs. 10 images Maize	-10.692	0.78	944	-13.678	$<$0.0001
20 images COCO vs. 20 images Maize	-10.324	0.78	944	-13.192	$<$0.0001
20 images COCO vs. 30 images Maize	-11.769	0.78	944	-15.039	$<$0.0001
20 images COCO vs. 40 images Maize	-12.402	0.78	944	-15.847	$<$0.0001
20 images COCO vs. 50 images Maize	-12.345	0.78	944	-15.748	$<$0.0001
30 images COCO vs. 40 images COCO	-3.721	0.79	944	-4.736	$<$0.0001
30 images COCO vs. 50 images COCO	-5.873	0.79	944	-7.475	$<$0.0001
30 images COCO vs. 10 images Maize	-10.737	0.78	944	-13.736	$<$0.0001
30 images COCO vs. 20 images Maize	-10.369	0.78	944	-13.250	$<$0.0001
30 images COCO vs. 30 images Maize	-11.815	0.78	944	-15.097	$<$0.0001
30 images COCO vs. 40 images Maize	-12.447	0.78	944	-15.905	$<$0.0001
30 images COCO vs. 50 images Maize	-12.391	0.78	944	-15.805	$<$0.0001
40 images COCO vs. 50 images COCO	-2.152	0.79	944	-2.739	$<$0.0001
40 images COCO vs. 10 images Maize	-7.016	0.78	944	-8.976	$<$0.0001
40 images COCO vs. 20 images Maize	-6.648	0.78	944	-8.495	$<$0.0001
40 images COCO vs. 30 images Maize	-8.093	0.78	944	-10.342	$<$0.0001
40 images COCO vs. 40 images Maize	-8.726	0.78	944	-11.150	$<$0.0001
40 images COCO vs. 50 images Maize	-8.669	0.78	944	-11.059	$<$0.0001
50 images COCO vs. 10 images Maize	-4.864	0.78	944	-6.223	$<$0.0001
50 images COCO vs. 20 images Maize	-4.496	0.78	944	-5.745	0.0001
50 images COCO vs. 30 images Maize	-5.942	0.78	944	-7.592	0.0001
50 images COCO vs. 40 images Maize	-6.574	0.78	944	-8.401	0.1598
50 images COCO vs. 50 images Maize	-6.518	0.78	944	-8.314	0.1868
10 images Maize vs. 20 images Maize	0.368	0.78	944	0.474	0.2230
10 images Maize vs. 30 images Maize	-1.077	0.78	944	-1.388	0.4552
10 images Maize vs. 40 images Maize	-1.710	0.78	944	-2.203	0.5115
10 images Maize vs. 50 images Maize	-1.653	0.78	944	-2.123	0.6974
20 images Maize vs. 30 images Maize	-1.446	0.78	944	-1.858	0.9309
20 images Maize vs. 40 images Maize	-2.078	0.78	944	-2.671	0.9984
20 images Maize vs. 50 images Maize	-2.021	0.78	944	-2.591	0.9993
30 images Maize vs. 40 images Maize	-0.633	0.78	944	-0.813	1.0000
30 images Maize vs. 50 images Maize	-0.576	0.78	944	-0.738	1.0000
40 images Maize vs. 50 images Maize	0.057	0.78	944	0.073	1.0000

Supplementary Table S8: P-values of lsmeans in the statistical analysis of model updating with the factor model:trainingsetsize

Contrast between models	Estimate	SE	df	t ratio	p value
10 images COCO vs. 20 images COCO	-6.52	0.79	944	-8.29	$<$0.0001
10 images COCO vs. 30 images COCO	-6.47	0.79	944	-8.24	$<$0.0001
10 images COCO vs. 40 images COCO	-10.19	0.79	944	-12.97	$<$0.0001
10 images COCO vs. 50 images COCO	-12.34	0.79	944	-15.71	$<$0.0001
10 images COCO vs. 10 images Maize	-17.21	0.78	944	-22.02	$<$0.0001
10 images COCO vs. 20 images Maize	-16.84	0.78	944	-21.52	$<$0.0001
10 images COCO vs. 30 images Maize	-18.29	0.78	944	-23.37	$<$0.0001
10 images COCO vs. 40 images Maize	-18.92	0.78	944	-24.17	$<$0.0001
10 images COCO vs. 50 images Maize	-18.86	0.78	944	-24.06	$<$0.0001
20 images COCO vs. 40 images COCO	-3.68	0.79	944	-4.68	$<$0.0001
20 images COCO vs. 50 images COCO	-5.83	0.79	944	-7.42	$<$0.0001
20 images COCO vs. 10 images Maize	-10.69	0.78	944	-13.68	$<$0.0001
20 images COCO vs. 20 images Maize	-10.32	0.78	944	-13.19	$<$0.0001
20 images COCO vs. 30 images Maize	-11.77	0.78	944	-15.04	$<$0.0001
20 images COCO vs. 40 images Maize	-12.4	0.78	944	-15.85	$<$0.0001
20 images COCO vs. 50 images Maize	-12.35	0.78	944	-15.75	$<$0.0001
30 images COCO vs. 40 images COCO	-3.72	0.79	944	-4.74	$<$0.0001
30 images COCO vs. 50 images COCO	-5.87	0.79	944	-7.48	$<$0.0001
30 images COCO vs. 10 images Maize	-10.74	0.78	944	-13.74	$<$0.0001
30 images COCO vs. 20 images Maize	-10.37	0.78	944	-13.25	$<$0.0001
30 images COCO vs. 30 images Maize	-11.81	0.78	944	-15.1	$<$0.0001
30 images COCO vs. 40 images Maize	-12.45	0.78	944	-15.91	$<$0.0001
30 images COCO vs. 50 images Maize	-12.39	0.78	944	-15.81	$<$0.0001
40 images COCO vs. 10 images Maize	-7.02	0.78	944	-8.98	$<$0.0001
40 images COCO vs. 20 images Maize	-6.65	0.78	944	-8.49	$<$0.0001
40 images COCO vs. 30 images Maize	-8.09	0.78	944	-10.34	$<$0.0001
40 images COCO vs. 40 images Maize	-8.73	0.78	944	-11.15	$<$0.0001
40 images COCO vs. 50 images Maize	-8.67	0.78	944	-11.06	$<$0.0001
50 images COCO vs. 10 images Maize	-4.86	0.78	944	-6.22	$<$0.0001
50 images COCO vs. 20 images Maize	-4.5	0.78	944	-5.75	$<$0.0001
50 images COCO vs. 30 images Maize	-5.94	0.78	944	-7.59	$<$0.0001
50 images COCO vs. 40 images Maize	-6.57	0.78	944	-8.4	$<$0.0001
50 images COCO vs. 50 images Maize	-6.52	0.78	944	-8.31	$<$0.0001
40 images COCO vs. 50 images COCO	-2.15	0.79	944	-2.74	0.1600
20 images Maize vs. 40 images Maize	-2.08	0.78	944	-2.67	0.1900
20 images Maize vs. 50 images Maize	-2.02	0.78	944	-2.59	0.2200
10 images Maize vs. 40 images Maize	-1.71	0.78	944	-2.2	0.4600
10 images Maize vs. 50 images Maize	-1.65	0.78	944	-2.12	0.5100
20 images Maize vs. 30 images Maize	-1.45	0.78	944	-1.86	0.7000
10 images Maize vs. 30 images Maize	-1.08	0.78	944	-1.39	0.9300
20 images COCO vs. 30 images COCO	0.05	0.79	944	0.06	1.0000
10 images Maize vs. 20 images Maize	0.37	0.78	944	0.47	1.0000
30 images Maize vs. 40 images Maize	-0.63	0.78	944	-0.81	1.0000
30 images Maize vs. 50 images Maize	-0.58	0.78	944	-0.74	1.0000
40 images Maize vs. 50 images Maize	0.06	0.78	944	0.07	1.0000

Supplementary Figures

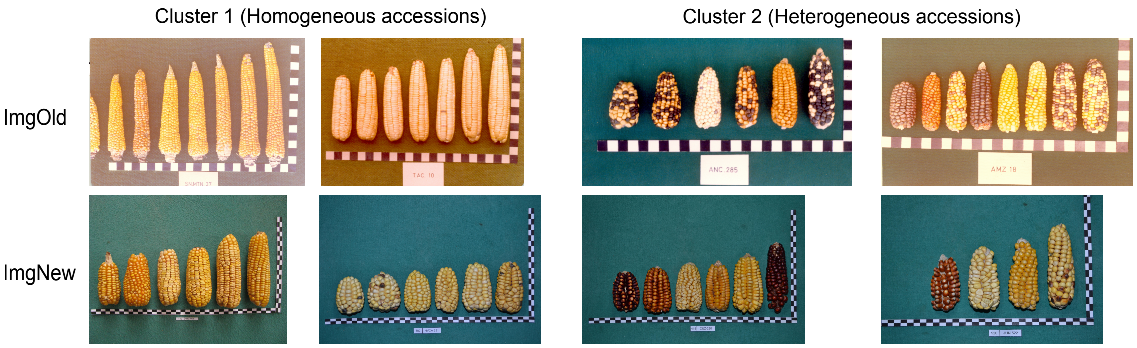

Supplementary Figure S1: Example images of homogenous and hetergenous genebank accessions identified by the multivariate clustering of extracted RGB colors and morphological traits.

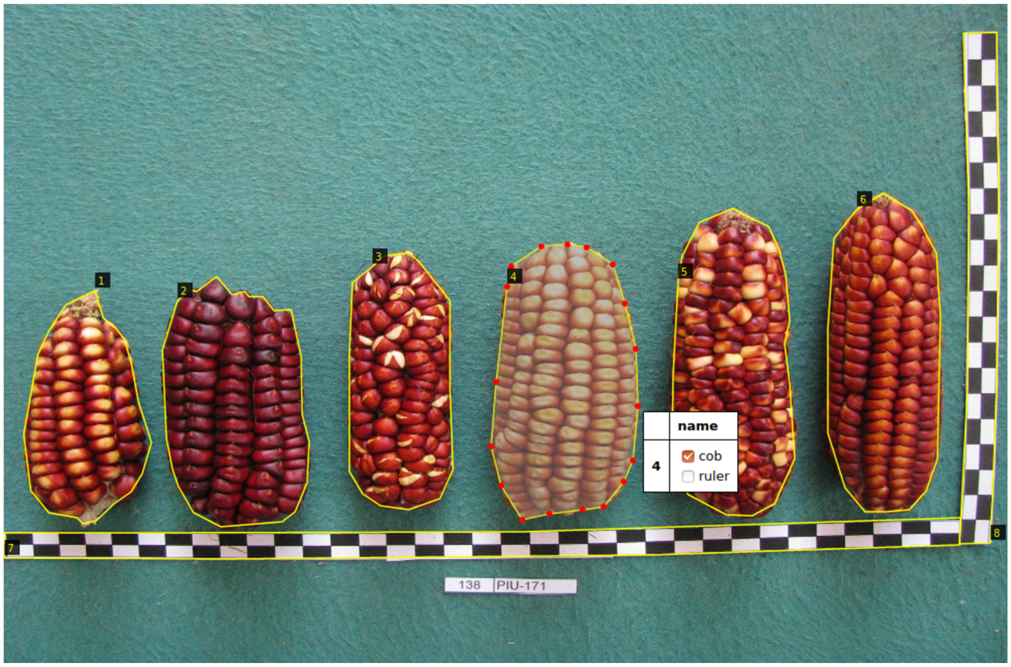

Supplementary Figure S2: Data annotation for *Mask R-CNN* . Polygons were drawn around each object. The ruler was split into the horizontal and the vertical part (to enable also ruler detection on images with only one ruler part) resulting in 2 annotated ruler objects. Finally, the classes of the objects were selected from a checkbox.

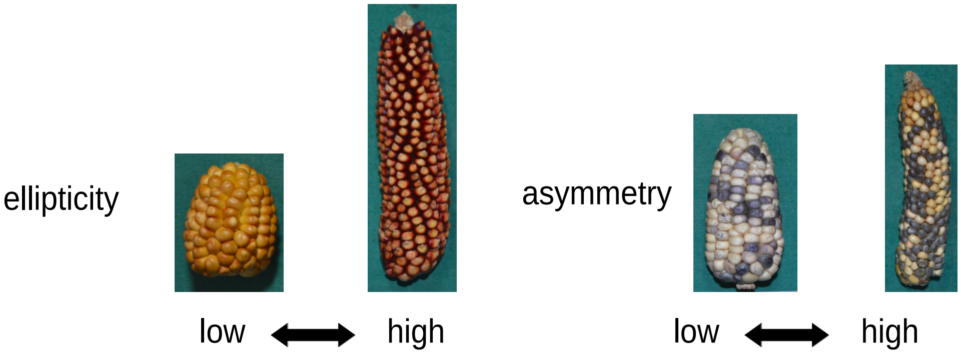

Supplementary Figure S3: Examples for maize cobs with high and low asymmetry and ellipticity, respectively. Cobs with a low asymmetry value have a very symmetrical morphometric shape, whereas for highly asymetrical cobs high asymmetry values are calculated. Similarly, cobs with a low ellipticity are rather round, whereas high ellipticity values are found in elliptical cobs. For further information regarding the calculation and interpretation of these morphometry values please refer to Ref. [**?**, **?**].

.

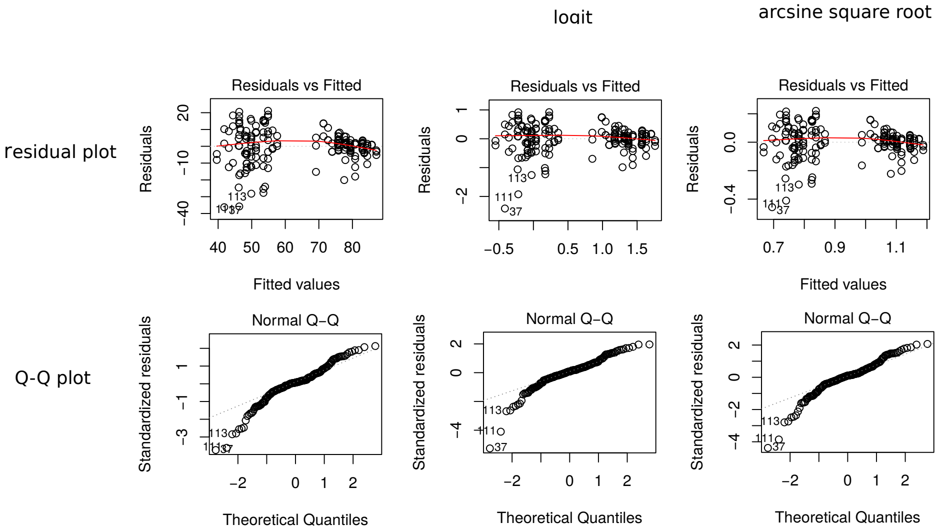

Supplementary Figure S4: Statistical analysis for *Mask R-CNN* parameter selection: Residual plots of different transformations. Logit transformation showed the best results in terms of heterogeneity of variance (residual plot) and normal distribution (QQ-plot) and was therefore applied in the analysis.

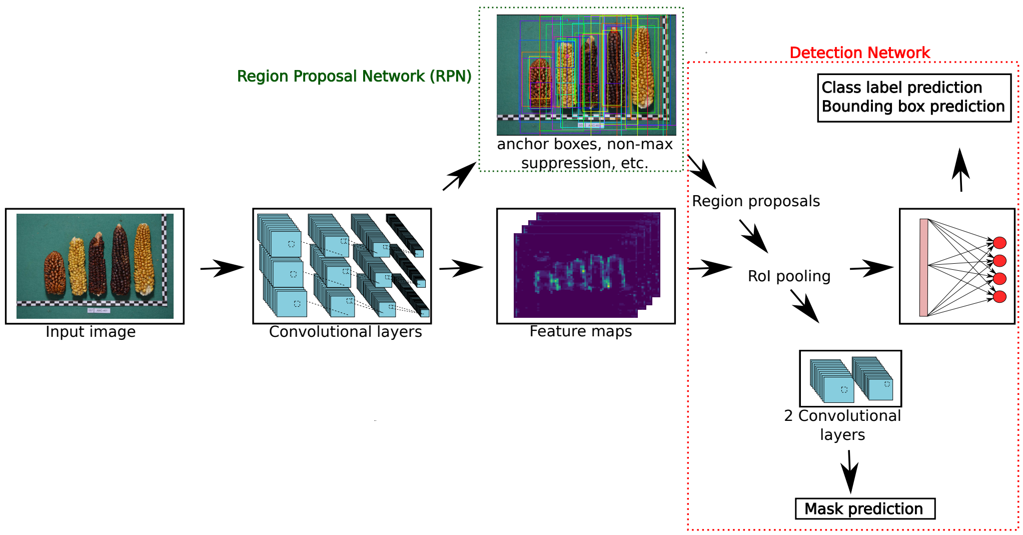

Supplementary Figure S5: Structure of Mask R-CNN visualized with cob images. Mask R-CNN with a block of 2 convolutional layers on top for binary mask prediction.

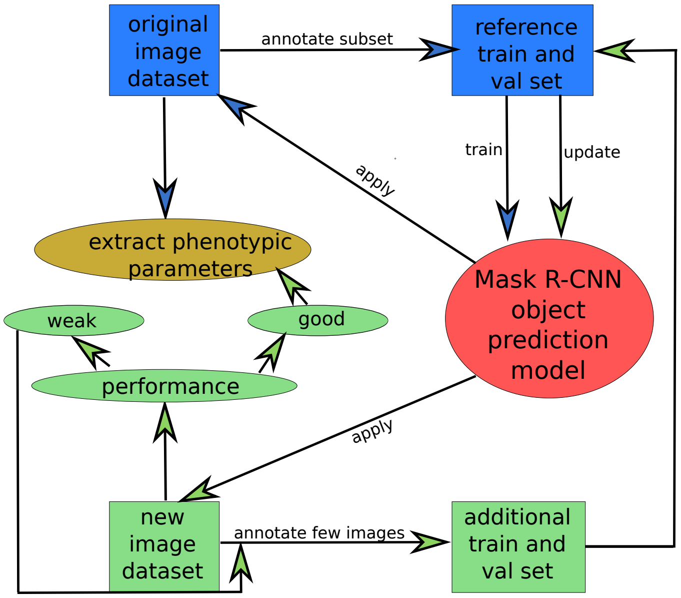

Supplementary Figure S6: Iterative updating scheme of *Mask R-CNN* for application on different datasets and subsequent extraction of phenotypic parameters. In case the model of *Mask R-CNN* does not perform well on a new dataset, the object prediction model is iteratively updated. Two options for the updating step are possible: Either some annotated images from the new dataset are added to the reference train and validation set to update the model (shown here) or the model is re-trained only on these new images.
